# Examining the evidence for best practice guidelines in supportive supervision of lay health care providers in humanitarian emergencies: A systematic scoping review

**DOI:** 10.7189/jogh.12.04017

**Published:** 2022-02-27

**Authors:** Nadeen Abujaber, Frédérique Vallières, Kelly A McBride, Greg Sheaf, Pia Tingsted Blum, Nana Wiedemann, Áine Travers

**Affiliations:** 1 Trinity Centre for Global Health, School of Psychology, Trinity College Dublin, College Green, Dublin, Ireland; 2 International Federation of Red Cross and Red Crescent Societies, Copenhagen, Denmark

## Abstract

**Background:**

Supervision is widely recognised as an important form of support for lay health service providers. However, guidance in appropriate supervision practices for task-shifting health interventions within the unique context of humanitarian emergencies is lacking. This review set out to identify empirically supported features of supervisory practices for lay health care providers in humanitarian emergencies, towards a stronger evidential basis for best practice in supportive supervision.

**Methods:**

In January 2021, six databases and five non-governmental organizations’ websites were searched for articles examining the effectiveness of supervision for health care interventions delivered by lay providers in humanitarian settings. The inclusion criteria for study selection were qualitative or quantitative primary studies, articles published in peer reviewed journals or technical reports and the availability of the studies in English. The outcomes of interest were client clinical outcomes, health service efficiency and sustainability, and lay health care providers well-being. All articles were independently reviewed by the first and last authors.

**Results:**

A total of 3371 articles were initially identified, with a total of 11 articles retained following the systematic screening process (two quantitative, four mixed methods and five qualitative studies). All studies generally reported positive impacts of supportive supervision on client outcomes, service sustainability, staff well-being and staff performance. Only four studies offered emotional support as part of supportive supervision. No studies evaluated the effect of supportive supervision on service efficiency. The narrative synthesis suggests significant challenges with providing supportive supervision, including excessive workloads, difficult supervisory relationships, geographic dispersion of lay providers, safety concerns, poorly trained supervisors, and lack of supervisory guidelines.

**Conclusions:**

More efforts are needed to prioritize supportive supervision in task-shifting frameworks and to ensure that supervision is regular, consistent and of high-quality, with well-trained and well-supported supervisors.

Widely recognised as foundational to any health system, the availability of skilled health workers is key to the delivery of safe and equitable health services. Unfortunately, the World Health Organization (WHO) projects that the current health workforce deficit will increase to approximately 18 million providers by the year 2030 [1]. Moreover, the distribution of health providers tends to be overly concentrated in urban centres, leaving health systems, disproportionally those in the low- and middle-income countries (LMICs) of sub-Saharan Africa and Southeast Asia [1], unable to cater to the medical needs of more rural catchment areas [2]. The global shortage, inequitable distribution, and skill-mix imbalance of health workers, therefore, remain key barriers to achieving universal health coverage (UHC).

Task-shifting is a practice that specifically addresses deficient supply and distribution of personnel, by cascading responsibility for less specialised tasks to providers with fewer formal qualifications [3]. Task-shifting therefore often makes use of ‘lay health workers’ also known as ‘lay health care providers’; individuals who are often from the community they are serving, who are not health care professionals, but are trained to provide certain health care interventions [4,5]. Lay health workers have been shown to effectively deliver a range of services, including health promotion and education, as well as diagnostics and therapeutics [6,7]. Specific examples include diagnosis and treatment of common childhood diseases (pneumonia, diarrhoea, and malaria), nutritional support, HIV education, testing and initiation of anti-retroviral therapies (ART), contraception counselling as well as mental health screening and education [8].

In 2011, the Global Health Workforce Alliance, WHO, International Federation for Red Cross (IFRC), United Nations International Children’s Emergency Fund (UNICEF) and United Nations High Commission for Refugees (UNHCR) published recommendations to expand the responsibilities of lay workers to include emergency care in humanitarian emergencies [9]. As stated by Miller et al. [10], lay providers “represent one of the most promising options for delivering basic healthcare services to populations affected by humanitarian emergencies”. To date, however, few studies have examined the effectiveness of task-shifting within emergency settings [10]. Therefore, less is known about the factors that contribute to lay health worker effectiveness within humanitarian contexts, where health care workers may be subject to deliberate violent targeting during conflicts or are themselves victims of natural disasters, resulting in further health worker flight, morbidity, and mortality [11,12]. Accordingly, providers that remain are expected to work in high stress conditions, often without basic resources and supports [11,13].

In their study in South Sudan, Kozuki et al. [14] found that lay providers, even those that had been internally displaced, were able to provide continued diagnostic and therapeutic interventions for childhood illnesses and families. Moreover, this care was often preferred by the patients, who felt it was safer than the formal health sector due to security risks [14]. Miller et al. [15] also reported similar findings across multiple settings where lay personnel provided maintained services despite the challenges of acute and protracted humanitarian crises, resulting in a faster recovery than settings that did not have a task-shifting mechanism in place. Ruckstuhl et al. [16] concluded that a programme where lay providers were responsible for diagnosing, managing, and treating malaria during civil and political unrest in the Central African Republic increased access to care for vulnerable and neglected populations and improved malaria surveillance. Another example from post-conflict Liberia demonstrated that training lay providers to deliver ART and psychosocial support to patients in rural settings who had no access to HIV care resulted in improved health outcomes and enhanced survival one year after the launch of this initiative [12].

Similar to task-shifting programmes implemented in non-humanitarian contexts, it stands to reason that lay health workers operating in humanitarian contexts also require that certain conditions be met to maximise the effectiveness of health services. To build strong networks between communities and health systems, increase healthy behaviour and ultimately, improve patient outcomes using task-shifting approaches, it is essential to have clearly defined roles, reasonable workloads with adequate resources, appropriate training, and supportive supervision [17]. Moreover, due consideration must be given to the unique experience of working within emergency contexts, including emotional exhaustion caused by high volumes of work and stressful conditions, as well as the trauma experienced by the beneficiaries they are serving [18]. Lay providers should further be formally recognised, respected, and integrated into the health system, as well as accepted and well-regarded by the communities they serve to facilitate their activities [8,17]. Delivery of maternal and child health services by lay providers, for example, was significantly hampered by weak support from the health system, inadequate resources, and mistrust from the communities during the West African Ebola outbreak [19]. Similarly, lay providers heavily involved in disaster relief services during the 2015 earthquake in Nepal cited poor training in disaster care, insufficient collaboration with aid agencies, and inadequate supervision as adversely impacting their efforts [20]. Indeed, supportive supervision is widely recognised as an important predictor of a range of performance-related outcomes among lay health providers [21], and as a vital resource to help harness the benefits of task-shifting frameworks and to protect the health and well-being of lay providers working within them [11].

Supportive supervision is defined as the mechanism of providing both technical and emotional assistance to staff on an ongoing basis [22]. Quality and consistent supervision should endeavour to ensure that lay workers are able to meet the challenges they face during task-shifting activities in the following ways: evaluating practices with suggestions for improvement and development; helping to solve clinical scenarios that go beyond the scope of lay providers’ experience and training; providing recognition and legitimacy to lay providers within communities and health systems; as well as monitoring for signs of burnout and providing emotional support and coping strategies [18,23-27].

Despite the documented advantages to supervision, Hill et al. [23] found that the supervision of task-shifting arrangements is often unavailable or of poor quality. Numerous studies from a variety of settings have demonstrated similar realities to those described by Hill et al. [23]: irregular or absentee supervision as well as demotivating or unsupportive supervisors who are poorly trained to provide knowledgeable and effective feedback [28-30]. For example, a study conducted in Ghana, found that less than 15% of lay providers reported feeling as though they could rely on their supervisors for support [30]. Ludwick et al. [31] found that ineffective supervision negatively impacted the performance of lay providers in task-shifting activities, consequently threatening treatment goals and health outcomes. Gaps in supportive supervision are even greater in humanitarian emergencies, where supervisors are fewer in number, inadequately trained, and lack the space and time needed to carry out their roles [32].

Taken together, and while task-shifting offers a promising approach to fill the gaps in health care service delivery in humanitarian emergencies; the lack of guidelines regarding appropriate supervisory practices in humanitarian contexts limits the effectiveness of services delivered by lay health providers. Accordingly, the Human Resources in Humanitarian Health (HRHH) Working Group-a team tasked with evaluating and addressing the global health workforce crisis - emphasised during the 2009 Humanitarian Action Summit, that task-shifting endeavours must be complemented by ongoing supervision from trained health professionals [12]. This review thus aims to identify those features of supervisory practices that are empirically supported for lay health providers working in humanitarian settings, with a view to informing the development and testing of best practice guidance for supportive supervision in emergency contexts.

## METHODS

### Eligibility criteria

The eligibility criteria for study selection were 1) qualitative or quantitative primary studies including some evaluation of the supervision of lay providers delivering a health intervention in a humanitarian context; 2) published in either peer reviewed journals or as technical reports; 3) available in English. The outcomes of interest were 1) client clinical outcomes, 2) health service efficiency and sustainability, and 3) lay health worker well-being. For the purpose of this review, the definitions of the abovementioned terms are found in **Table 1**.

**Table 1:** Definition of terms

Term	Definition
Lay health worker/lay health care provider	Defined as a ‘member of the community who has received some training to promote health or to carry out some health care services, but is not a health care professional’ [5]
Humanitarian context	Settings of natural disaster, armed conflict, complex emergencies and their aftermath, political crises, and disease outbreaks such as Ebola and COVID-19
Client clinical outcomes	Defined as any ‘measurable changes in health, mental health, function or quality of life’ for those receiving health services [33]
Health service efficiency	Defined as ‘how well health care resources are used to obtain health improvements’ [34]
Health service sustainability	Defined as the ‘the long-term ability to mobilize and allocate sufficient and appropriate resources (manpower, technology, information and finance) for activities that meet individual or public health needs/demands’ [35]
Lay health worker well-being	Defined as the state 'which allows individuals to realise their abilities, cope with the normal stresses of life, work productively and fruitfully, and make a contribution to their community’ [36]

### Search strategy

Key search terms were obtained from related research and the full search strategy is available in Appendix S1 in the **Online Supplementary Document**. On the 11th of January 2021, a search was initiated and completed via the six following databases using the abovementioned terms: Web of Science, Medline, Embase, Cinahl, Psychinfo and PDAT. Results were uploaded into the systematic review application Covidence [37] and duplicates were removed. The first and last authors (NA and AT) then independently screened article titles and abstracts against the inclusion criteria, removing any articles that failed to meet all the criteria. Full texts were then screened independently by the two reviewers against the inclusion criteria. A third reviewer (KM) was consulted to analyse and resolve any conflicts in screening decisions between the first and last authors. Any remaining articles were then brought forward for data extraction. In addition to the abovementioned database searches, the websites of WHO, Médecins Sans Frontiers (MSF), United Nations International Children’s Fund (UNICEF), United Nations High Commission for Refugees (UNHCR) and International Federation for the Red Cross (IFRC) were searched using the following terms: ‘supervision’, ‘supportive supervision’, ‘supervision of lay providers’, ‘supervision of task shifting’, ‘supervision in humanitarian emergencies’ and ‘supervision in conflict’.

### Data extraction and analysis

The first author, with the assistance of the last author, extracted the following pertinent data from included studies: author year and location, study purpose, study design, participant characteristics, outcomes of interest, description of supervision (ie, type, duration, frequency, goals), evaluation of supervision, and other relevant study findings and challenges identified. Narrative synthesis was then applied to present and analyse the findings, as an appropriate approach to apply when reviews cannot be synthesised statistically [38]. The critical appraisal skills programme (CASP) checklists [39,40] were used to systematically evaluate the quality of the randomized controlled trial [41] and the qualitative studies [2-45]. The JBI critical appraisal checklist [46] was used to examine the selected cross-sectional study [47] and the Mixed Methods Appraisal Tool (MMAT) [48] was utilised to evaluate the four mixed methods studies [14,49-51].

## RESULTS

**Figure 1:**
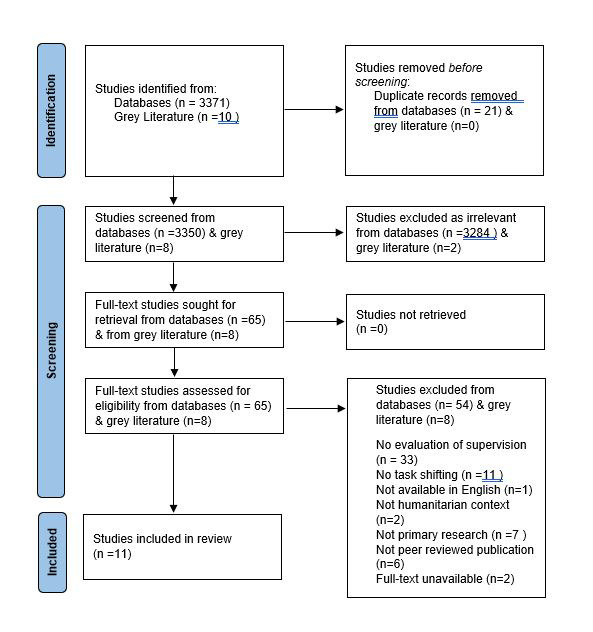
PRISMA flow diagram for study identification, screening and inclusion from databases and grey literature [52].

The initial search yielded 3371 articles. Of these, 21 were identified as duplicates and were removed, and 3284 articles failed to meet the inclusion criteria. The full texts of the remaining 65 studies were then screened, resulting in the removal of an additional 54 articles, and the retention of 11 articles for data extraction. The search of the NGO and UN databases identified a further 10 articles, but all were removed for not meeting the inclusion criteria. This process is depicted in the PRISMA flow diagram, as shown in **Figure 1****.**

### Description of included studies

Of the 11 articles included in this review, two were quantitative, five were qualitative and four employed mixed methods. Eight articles clearly described the supervisory parameters used including the type of supervision (peer, group, individual, etc.), whereas the supervision format was unclear in the remaining three articles [42,43,47]. More than half of the included research defined the goals of supervision [14,32,41,44,49,50]. Although no study directly compared different types of supervision and only one study [44] examined a training model with and without supervision, all studies emphasised the importance of supportive supervision to enhance staff well-being [32,44,45,47], to improve client outcomes [42,43,49,50], to increase service sustainability [14,41,43,44,51], and advance staff performance and competence [41-43]. No study explicitly evaluated the impact of supervision on service efficiency.

### Quality assessment of included studies

The majority of the included studies used primary data. Some studies analysed secondary data such as policy documents [32], District Health Information Software data [51], field reports [51], and supervisory records [45]. All the included studies evaluated supervisory practices in some capacity. While two studies [14,51] focused more heavily on the quantity of supervision sessions received before, during, and after humanitarian emergencies, attempts were also made to explore supervision through the experiences of supervisors and lay supervisees. Aldamman et al. [47] used a cross-culturally validated scale, the Perceived Supervision Scale [21], to evaluate supportive supervision. Rahman et al. [41] used the Enhancing Assessment of Common Therapeutic Factors (ENACT) rating scale [53], which though does not directly evaluate supervision, was used to evaluate the impact of supervision on the competence of lay providers. Six studies used key informant interviews and/or focus group discussions with supervisors and supervisees to evaluate the process challenges of supervision [14,32,42-44,51].

### Quality appraisal

Rahman et al. [41] fulfilled the needed criteria for quality but had two areas of potential weakness: a 25% attrition rate not accounted for and the absence of blinding for researchers and participants. The qualitative studies met the necessary criteria for quality apart from Wong and Leung [45], which had a suboptimal research design, limited data analysis and the absence of explicit ethical considerations discussed. Aldamman et al. [47] was awarded a seven out of a total eight points and all four mixed methods studies fulfilled the needed criteria for the quality assessment. The complete details of these quality analyses are depicted in **Tables 2-5**.

**Table 2:** CASP Checklist applied to the randomized controlled trial [41]

CASP Randomized Controlled Trials Checklist [40]	Focused research question	Participant randomisation	Intention to treat analysis used	Blinding	Study groups similarity	Equal treatment of groups	Comprehensive reporting	Confidence interval use	Benefits outweigh risks	Applicable results	Valuable research
Rahman et al., 2019 [41]	Yes	Yes, by independent team	No	Data analysis only	Yes	Yes, apart from intervention	Yes	Yes	Yes	Yes	Yes^12^

CASP – Critical Appraisal Skills Programme

**Table 3:** CASP checklist applied to the qualitative studies [32,42-45]

Casp qualitative studies checklist [39]	Clear research question	Appropriate methodology	Appropriate study design	Appropriate recruitment strategy	Data collection addressed research issue	Relationship between researcher and participants explored	Ethical issues considered	Rigorous data analysis	Clear findings	Valuable research
Raven et al., 2020 [32]	Yes	Yes	Yes	Yes	Yes	No	Yes	Yes	Yes	Yes
Miller et al., 2020 [42]	Yes	Yes	Yes	Yes	Yes	No	Yes	Yes	Yes	Yes
Horn et al., 2019 [43]	Yes	Yes	Yes	Yes	Yes	No	Yes	Yes	Yes	Yes
McLean et al., 2015 [44]	Yes	Yes	Yes	Yes	Yes	Yes	Yes	Yes	Yes	Yes
Wong and Leung, 2020 [45]	Yes	Yes	Yes	Yes	No	No	No	Limited	Yes	Yes

CASP – Critical Appraisal Skills Programme

**Table 4:** JBI Critical Appraisal Checklist applied to the cross-sectional study [47]

JBI Critical Appraisal Checklist Cross-Sectional studies [46]	Clear inclusion criteria	Detailed description participants and setting	Exposure measurement valid and reliable	Standard measurement criteria	Confounding factors identified	Strategy to address confounding factors	Outcome measurement valid and reliable	Appropriate statistical analysis
Aldamman et al., 2019 [47]	Yes	Yes	Yes	Yes	Yes	No	Yes	Yes

JBI-Joanna Briggs Institute

**Table 5:** MMAT criteria applied to the mixed methods studies [14,49-51]

MMAT [48]	Clear research question	Data collection addresses question	Clear design rationale	Study components integrated	Adherence to quality criteria
Kozuki et al., 2018 [14]	Yes	Yes	Yes	Yes	Yes
Murray et al., 2014 [49]	Yes	Yes	Yes	Yes	Yes
Magdison et al., 2015 [50]	Yes	Yes	Yes	Yes	Yes
Shah, Miller and Mothabbir, 2019 [51]	Yes	Yes	Yes	Yes	Yes

MMAT – Mixed Methods Appraisal Tool

### Evaluation of supervision practices

The results of all included studies are presented in **Table 6** and outlined in detail in the following narrative synthesis.

**Table 6:** Data extraction table

Authors, year, context	Study design	Participants description	Supervision description	Supervision evaluation	Supervision challenges identified
Kozuki et al., 2018 [14], South Sudan	Mixed methods	1.CBD (n = 3 FGDs): all F, 20-40 clients each. 2.Supervisors (n = 2 FGDs +3 IDIs)-IRC staff. 3.Policy makers (n = 10 IDIs). 4.Community leaders (n = 4 FGDs). 5.Program Implementers (n = 5 IDIs) : IRC field staff	In-person supervision, monthly. 15-20 supervisees/supervisor. *Goals:* Supply Inventory and distribution, link to formal health system, performance evaluation	Supervision shifted towards rescue efforts for displaced CBDs.	Insecurity disrupted supervision, transportation challenges
Raven et al., 2020 [32], Sierra Leone, Liberia and DRC	Qualitative + policy document review.	1. CHWs (Kenema, n = 8: 4F/4M) and Bonthe, n = 7: 5 F/2M). 2. Decision-makers (Sierra Leone, n = 9:7F/2M), Liberia (n = 10:2F/8M), DRC (n = 8: 3F/5M)	Sierra Leone: 1. Peer supervisor: Monthly. Goal: observation, coordination. 2. Health unit manager: monthly. Goals: training, troubleshooting, supplies. 3. District focal person: training, visits, reports. DRC: 1. CHW group chair: monthly. 2. Facility head nurse: monthly. Goals: training, troubleshooting. Liberia: 1. CHSS: in person, 10 CHWs each. 2. Facility manager: Checks reports, f/u issues, reports to district health	Sierra Leone: support improved motivation and service delivery. DRC: Supervision identified areas for improvement and strengths, support and resources mobilized. Liberia: Not evaluated	Transportation challenges, overloaded schedules, poor supervisory training, strained relations between supervisor and CHWs, absent standardization of performance evaluation measures
Rahman et al., 2019 [41], Pakistan	Quantitative	1.LHWs (n = 80)-MOH trained, avg. age 35.5 years, avg. work years 12. Two LHW groups are similar. 2.SMT (n = 2). 3. NST (n = 2). 4. LHS (n = 8)	1. Conventional Method: *Training:* SMTs->LHWs (5 d). *Supervision:* LHS and SMT supervised LHWs (15-20 LHWs/LHS), group, monthly. *Goal:* role plays + feedback. 2. Cascade training/supervision model: *Training:* SMT->NST->LHS->LHS->LHWs (5 d each). *Supervision:* 3 levels of supervision: LHS and LHWs (monthly, in person, group), NST and LHS (monthly, in-person group), SMT and NST (monthly, remote, individual). Goals: feedback, role plays, intervention fidelity, workload check	Monthly supportive supervision key to improve LHW competency. TACTS: LHW Competency achieved remotely: advantage in LMICs with limited specialists.	Limited availability of stable internet availability in conflict zones, 25% sample lost to follow up
Miller at al., 2020 [42], Yemen	Qualitative	1. Policy makers (n = 2). 2. Program Implementers (n = 10). 3. Health workers (n = 4). 4. CHW supervisors (n = 4). 5. Community leaders (n = 3). 6. CHWs (n = 2 FGDs)	Not described	Limited consistent, quality supervision	Distance, safety concerns, competing demands, poor supervisory training
Horn et al., 2019 [43], Sierra Leone and Liberia	Qualitative	1. Trainers (n = 23: 10M/13F, 15 Sierra Leone, 18 Liberia, Avg. work 1-26 years). 2. Lay providers (n = 36: 23M/13F, 17 Sierra Leone, 19 Liberia. Avg. work 1-16 years). 3. Program managers (n = 14: 6 Sierra Leone, 8 Libéria)	Not described apart from trainers supervising providers	Supervision necessary for fidelity and technical assistance, but variable in quality and consistency	Short training course, limited experiences
McLean et al., 2015 [44], Haiti	Qualitative	Phase 1: IDI (n = 18). Phase 2: IDI (n = 2) + observational data (n = 14 CHWs). Phase 3: 1st FGD (n = 7). 2nd FGD (n = 8)	Apprenticeship model: 1-week daily sessions, observation followed by practice of learned skills under supervision. Goals: debriefing, troubleshooting, brainstorming.	Training supervision: strongest predictors of behaviour change. Should offer emotional support. Phase 2: improved knowledge re. MHPSS topics but minimal retention over time. Phase 3: 2 y MHPSS job retention. Improved knowledge and confidence	Short training course
Wong and Leung, 2020 [45], China	Qualitative	1. Supervisors (n = 16). 2. Counsellors (n = 8 teams), 38 sessions completed	Group supervision for volunteers. Individual supervision for extra support. Goals: Emotional support, burnout prevention	Best structure for Supervision: Sensitization-Emotional Support-Scanning-Continual Education-Repeated warnings against burnout. Advantage online platform: decrease power and status barriers.	Not described
Aldamman et al., 2019 [47], Sudan	Quantitative	Volunteers (n = 409, 182 F/223 M) Avg. volunteer years = 6 years, Avg. work = 11.14 h/week	Not described	Direct relationship between supervision and mental health outcomes. Good supervision contributor to well-being	Not described
Murray et al., 2014 [49], Iraq and Thailand	Mixed methods	1. Lay counsellors (Iraq: n = 12: MOH, experienced. Thailand: n = 20: 16 Inexperienced). 2. Supervisors (Iraq: n = 2 MDs, Thailand: n = 3: MD x1, 2 without training). 3. Clients (n = 34: 12 Iraq, 22 Thailand)	Apprenticeship model: Staggered training with in-person supervision. *Activities:* Role plays + feedback in person or remotely. *Supervision:* weekly. *Goal:* Treatment fidelity. Supervisors got remote supervision by US trainer.	Role plays showed incorporation of training into performance with treatment fidelity achieved. Improved weekly symptom scores in both settings.	Restricted time, transportation issues, gender role barriers
Magdison et al., 2015 [50], Iraq	Mixed methods	1. CHWs (n = 11). 2. Study MD (n = 1). 3. TK (n = 1, BATD training. 4. BATD experts (n = 2, US based)	1.CHW: weekly supervision from BT, in person or remote. Goal: review cases and troubleshoot. 2.BT: weekly supervision from US BATD expert, remote. Goals: challenges, technical issues.	BATD treatment by CHWs decreased depression symptoms and improved functioning vs control	Technical difficulties, language barriers
Shah, Miller and Mothabbir, 2019 [51], Bangladesh	Mixed methods	1. CHCPs (n = 44, MOH, >12 grade, 3-mo training) 2. VD (n = 7, MDs, refer complications to MOH facilities). IDI (n = 28: 12 CHCPs/5 VDs, 8 supervisor, 3 policy makers. FGD (n = 13: supervisors + community leaders)	CHCPs and VDs supervised by MDs from sub-district health centre in person, monthly.	51% supervision reduction with flooding. 100% supervision achieved in non-flooding. After flooding, supervision recovery rate to 74%-85%.	Transportation challenges with flooding

CBD – community based distribution, LHW – lady health workers, MOH – Ministry of Health, SMT – specialist master trainer, NST- non-specialist trainer, LHS – lady health supervisor, CHCP – community health care provider, VD – village doctor, CHSS – community health service supervisors, CHW – community health workers, TL – CHW clinical supervisor, BT – bilingual therapist, BATD – Behavior Activation Treatment for Depression, TACTS – Technology Assisted Cascade for Training and Supervision, IDI – in-depth interviews, FGD – focus group discussions, IRC – International Rescue Committee, DRC – Democratic Republic of Congo

#### I. Supervision descriptions

- *Type of supervision*: Eight studies described the supervision provided, some utilising a mix of different modalities. Four articles delineated individual face-to-face supervisory structures between lay providers and their appointed supervisor [14,32,50,51] with a fifth mobilising individual supervision for those identified as needing additional support [45]. Two studies used individual supervision but in a remote capacity [41,50]. Group supervision was another popular option, especially in resource limited settings, with multiple lay providers supervised by one designated supervisor [32,41,45]. Peer supervision was described in one setting in Sierra Leone, where community health workers (CHWs) supported each other, supervised by a CHW who had received additional supervisory training [32]. Murray et al. [49] and McLean et al. [44] used an apprenticeship model [54], where training sessions were combined with one-on-one intensive supervision. A cascade supervision model was introduced by Rahman et al. [41], whereby a specialist trains a supervisor, who then trains lay providers. Three studies also provided supervision for supervisors, delivered remotely on an individual basis by a foreign expert [41,49,50].
- *Frequency of supervision*: The majority of supervision occurred monthly apart from the sessions described by Murray et al. [49] and Magdison et al. [50], which occurred on a weekly basis.
- *Supervisor to supervisee ratios*: The following supervisor to supervisee ratios were described: 1 to 15-20 [14,41], 1 to 10 [32,49] and 1 to 1 for the apprenticeship models [44,49].
- *Supervision goals:* Six articles delineated aims for the supervisory sessions under the following themes: troubleshooting challenges [41,44,50], verifying intervention fidelity and technical guidance [14,32,41,43], assessing workload [41], providing emotional support [41,45], linking lay providers to the formal health system and coordinating their activities [14,32] as well as checking and supplementing inventory and supplies [14,32].

#### II. *Supervision findings*

The included studies reported the impact of supportive supervision on the following parameters: client clinical outcomes, health service sustainability, and lay health worker wellbeing. None of the studies evaluated health service efficiency explicitly, but there was information about the impact of supervision on lay health worker performance.

- *Client clinical outcomes:* Using an apprenticeship model for training and supervision, Murray et al. [49] found that mental health services provided by lay counselors resulted in a decrease in symptoms of depression, anxiety, and post-traumatic stress disorder (PTSD) in survivors of torture and violence. Similar results were noted by Magdison et al. [50] when CHWs under supervision provided behavioral activation treatment for depression (BATD) to clients in Kurdistan, Iraq.
- *Health service sustainability:* After engaging lay providers in a training course to provide psychological care to those suffering from Ebola-related trauma, Horn et al. [47] found that when supervision was inconsistent, service delivery was interrupted and erratic. McLean et al. [44] noted that supervision was the strongest factor for persistent behavior modification. When supervision and training was provided to three lay providers via an apprenticeship model, not only did this improve competence and knowledge in shorter term, but these providers were still incorporating what they had learned into their practice two years later, unlike the group of lay providers who were trained without supervision. Rahman et al. [41] noted that in fragile contexts with limited resources, remote supervision yielded similar lay provider competence levels as in-person supervision, thus enhancing the potential for improved service sustainability at lower costs.
- *Staff wellbeing*: Aldamman et al. [47] found that supportive supervision, in combination with organizational and team support, was positively associated with mental wellbeing, and negatively associated with adverse mental health symptoms of anxiety, depression and perceived stress. In addition, Raven et al. [32] documented the experiences of lay providers and supervisors in Sierra Leone, Liberia, and Democratic Republic of the Congo (DRC) to highlight the ways CHWs should be supported in humanitarian settings. CHWs identified supervision, particularly one that is respectful and appreciative, as a motivating factor for their work. When they did not receive supervision or the supervisory relationship was tense, CHWs described feeling neglected and demoralised. McLean et al. [44] described an improvement in the perceived self-confidence of the three lay providers supervised using the apprenticeship model. McLean et al. [44] also concluded that supportive supervision should include emotional support for supervisees after many participants remarked on significant psychological stressors resulting from and affecting their work. This was echoed by Wong and Leung [45] who also emphasised that supervision should provide emotional support.
- *Staff performance:* Both Raven et al. [32] and Murray et al. [49] discussed how supervision was key to identifying areas in need of development and capacity building to ultimately improve the service delivery and performance of lay staff.

#### III. Identified challenges of supervision

- *Lack of standardization of supervision guidelines:* A challenge identified by supervisors in the study by Raven et al. [32] was the lack of standardized benchmarks to evaluate CHWs and their work, especially when a CHW was performing poorly. Supervisors reported relying on technical indicators such as reports given the lack of alternative guidelines. Furthermore, the supervisory relationship was often described as a hierarchical system rather than one of mutual trust and collaboration, with some supervisors viewing CHWs as competition for their jobs [32]. Magdison et al. [50] also found that the goals of supervision were often poorly defined, resulting in mismatched expectations between supervisors and supervisees.
- *Limited training and support for supervisors:* Miller et al. [42] noted that often supervisors receive no formal training in supervision and that this limited knowledge impinged on their ability to optimally guide the development and performance of CHWs.
- *Heavy workload and poor prioritization for supervision:* Time was often not allocated for supervision and supervisors had many competing demands, decreasing the frequency of their supervisory activities [42]. This was also found in Liberia, where one key informant reported that even though supervisors were expected to visit their supervisees twice per month, overloaded work schedules hindered some supervisors from attending even monthly meetings [32].
- *Logistical barriers:* Distance and transportation challenges were cited by both Kozuki et al. [14] and Miller et al. [42] as interfering with supervision. Supervisors reported having to travel as many as eight hours, often on foot, to reach their supervisees. During acute crises, destroyed roads and security concerns made these journeys nearly impossible, thereby limiting the consistency of supervision provided [14,42]. In addition, Magdison et al. [50] found that language and technical difficulties were also barriers interfering with the remote supervision provided by foreign supervisors.
- *Culture and gender roles:* Murray et al. [49] found that mixed gender supervisory pairings were not suitable in the more conservative context of Iraq without appointing a second supervisor that matched the gender of the supervisee. Female supervisors were also limited from travelling, especially during times of crisis, without a male escort.

## DISCUSSION

This systematic scoping review set out to identify the features of supervisory practices of lay health care providers in humanitarian emergencies empirically supported to improve client clinical outcomes, improve health service efficiency and sustainability, and enhance lay health worker well-being. Despite results emphasising the importance of supervision of lay providers in humanitarian emergencies, the evidence for best practice(s) within such contexts remains relatively scarce. For example, even though both Murray et al. [49] and Magdison et al. [50] demonstrated a significant decrease in the psychiatric symptoms of clients who had received treatment from lay providers under close supervision, both study results should be interpreted with caution. First, improvements in outcomes for Magdison et al. [50] were in comparison to waitlist controls, limiting the conclusions that can be made about the effectiveness of supervision, but nevertheless suggesting that supervised intervention is better than no intervention at all. Similarly, Murray et al.’s [49] pilot study lacked a control group. Taken together, results point to the need for future research comparing interventions delivered by supervised lay providers vs those that are unsupervised.

Moreover, there remains a need to identify which supervisory practice(s) are most effective in improving client clinical outcomes in humanitarian settings. Murray et al. [49] described using an apprenticeship model for supervision with weekly, in-person, or remote individual supervision for lay providers, with time spent in the field providing direct observation and feedback. Supervisors also received remote support and supervision from foreign experts. This latter supervisory format, with its emphasis on supervisor training and support, had previously been proposed by Murray et al. [54] as a particularly useful method for training inexperienced lay providers and supervisors in LMICs. However, and despite most humanitarian emergencies taking place in LMICS, Murray et al. [54] did not specifically focus on humanitarian contexts. Further research is therefore warranted to examine whether the apprenticeship model is the most appropriate and feasible modality to use in fragile states.

Though this systematic scoping review aimed to identify the impact of supervision on health service efficiency, none of the included studies assessed this specific outcome. However, the influence of supervision on service sustainability was examined by several articles. Horn et al. [43] found that supervision was qualitatively reported as a key factor to ensure continued service delivery, especially given the brevity of their training sessions. They reported that “where supervision was not possible, the PFA [psychological first aid] approach became diluted and confused” [43]. This finding was also reported by McLean et al. [44] who found that inexperienced lay providers, trained and supervised using an apprenticeship model, continued to provide MHPSS services even two years later, compared to a prior group who had been taught without supervision. In humanitarian contexts, where lay providers are often trained rapidly and briefly to provide services, ongoing supervision and support is thus thought to be positively associated with sustained service delivery.

In terms of the effect of supervision on lay health worker well-being, only four of the eleven studies included emotional support to lay providers as part of their supervision package. This is despite the widespread recognition that lay providers operating in humanitarian emergencies are at high risk for burnout, secondary traumatisation, and poor service delivery [18]. McLean et al. [44] reported that “during post-training interviews, participants cited family and financial problems and related personal emotional distress as barriers to engaging in [mental health and psychosocial services] MHPSS services for others”. Likewise, lay providers who had received emotional support from their supervisors reported improved motivation, self-confidence, as well as decreased depression, anxiety, and stress [32,44,47]. Though their findings were not empirically evaluated, Wong and Leung [45] highlighted what they considered to be key features of emotional support during supervision: mood assessments, education regarding self-care and burnout, promoting optimism and self-efficacy, as well as building a relationship based on trust and collaboration. Finally, Rahman et al. [41] evaluated their supervisees’ workload, and while they did not measure the impact of workload on well-being, the act of monitoring workload has previously been shown to help prevent supervisees’ from being overburdened, thereby decreasing their risks for burnout and improving their mental health [55].

Though staff performance was not part of this review’s main research objectives, three studies found that supervision improved staff competence, knowledge, and performance [32,41,49]. McLean et al. [44] further provided some evidence of improved staff knowledge and competence among those who had received supervision, compared to those who had not. One must consider however, that these groups differed in several ways including in size, demographics, experiences, and timing of intervention, limiting their comparability. Future research dedicated to comparing lay worker performance measures across those with and without supervision within emergency contexts should therefore compare groups with similar characteristics.

The absence or presence of supervision is not the only important factor to consider when evaluating the impact of supervision on staff, client, and service parameters. The quality of supervision provided is also essential, whereby suboptimal supervision has been shown to be as detrimental as no supervision [56]. Accordingly, the WHO highlights three essential areas for effective supervision, or “the three main Rs of an effective supportive supervision system”, as the right supervisors, the right tools, and the right resources [56]. Despite this recommendation, and consistent with previous studies [23,28-30], studies included in this review also found that supervision was often irregular and/or unsupportive. For example, distance, transportation challenges, and security risks were noted to impinge on the consistency of supervision provided [14,42]. Likewise, heavy workloads and competing demands resulting in absentee supervision were also reported [32]. Several studies described 15 to 20 supervisees assigned to one supervisor as excessive, consistent with the recommended number of six supervisees per supervisor reported elsewhere [57]. When coupled with the logistical challenges of distance and transportation in a humanitarian crisis, supervisors were not able to meet with each of their assigned supervisees regularly.

In addition to the frequency and consistency of supervision, some studies reported demotivating and unsupportive supervisory alliances. In one study, supervisees noted tension with their supervisors due to supervisees being viewed as competition [32]. They also described experiencing negative attitudes and behaviours from their supervisors, negatively impacting their work [32]. Another study discussed that supervisors were often inadequately trained for supervision, making supervisors poor sources of effective feedback and knowledge [42]. These findings demonstrate that further efforts are needed to prioritise and standardise the supervision of lay providers delivering health care in humanitarian emergencies by selecting the most qualified supervisors (or training potential supervisors properly), investing in the tools and resources to facilitate the success of supervision, and choosing a supervisory format based on the needs of the supervisees and with due consideration for their cultural context. For example, in certain contexts, it is important to consider whether a mixed-gender supervisory pairing is suitable [49]. Flexibility with, among other things, the format of supervision is also necessary within humanitarian contexts, where conditions can change acutely. The use of remote supervision may therefore be more beneficial in these circumstances. As demonstrated by Rahman et al. [41] technology-assisted supervision produced similar supervisee competence as traditional in-person supervision, at lower costs. Using an online platform could therefore help to circumvent some of the logistical barriers of distance, transportation, and security concerns characteristic of humanitarian emergencies, as well as increase the ability to scale-up services provided by lay providers in resource-limited settings.

### Limitations

The current review is not without limitations. First, only studies conducted in English were included in the review. Since humanitarian emergencies tend to occur predominantly in LMICs, where English is not necessarily the primary language, key information regarding supervision could have been missed. In addition, none of the studies systematically evaluated supervision against a control, nor, with the exception of Rahman et al. [41], did they compare different types of supervision modalities to one another.

## CONCLUSIONS

While the articles retained in this review generally support the use of supportive supervision for improved client clinical outcomes, health service sustainability, lay health worker well-being and lay health worker performance in humanitarian emergencies, none of the studies explicitly evaluated the effect of supervision on health service efficiency. In dynamic fragile states, where effective and expedient health care services are paramount and resources are limited, evaluating the relation between supervision and service efficiency therefore represents an important area for future research. Moreover, future research should aim to identify which supervisory practice(s) are most effective to achieve these outcomes within humanitarian contexts. Finally, the results of this review reiterate the challenges to providing consistent and motivating supervision within complex humanitarian contexts. More efforts are therefore needed to prioritise supportive supervision within task-shifting frameworks, and to provide the resources, tools, and appropriately trained supervisors required for supervision to take place within humanitarian emergencies. Standardised guidelines for how to best supervise lay providers in humanitarian emergencies would represent an important step towards these efforts.

## Additional material


Online Supplementary Document

## References

[1] World Health Organization. Framing the health workforce agenda for the Sustainable Development Goals: biennium report 2016–2017. Geneva: World Health Organization; 2017.

[2] World Health Organization. Working Together for Health: The World Health Report 2006. Geneva: World Health Organization; 2006.

[3] World Health Oorganization. PEPFAR and UNAIDS. Task shifting: rational redistribution of tasks among health workforce teams: global recommendations and guidelines. Geneva: World Health Organization; 2007.

[4] Barnett ML Lau AS Miranda J Lay Health Worker Involvement in Evidence-Based Treatment Delivery: A Conceptual Model to Address Disparities in Care. Annu Rev Clin Psychol. 2018;14:185-208. 10.1146/annurev-clinpsy-050817-084825 29401043 PMC5940491

[5] Lewin S Munabi-Babigumira S Glenton C Daniels K Bosch-Capblanch X van Wyk B Lay health workers in primary and community health care for maternal and child health and the management of infectious diseases. Cochrane Database Syst Rev. 2010;3:CD004015. 10.1002/14651858.CD004015.pub3 20238326 PMC6485809

[6] Lehmann U, Sanders D. Community health workers: what do we know about them? The state of the evidence on programmed, activities, costs and impact on health outcomes of using community health workers. Available: https://www.who.int/hrh/documents/community_health_workers.pdf. Accessed: 15 May 2021.

[7] Gilmore B McAuliffe E Effectiveness of community health workers delivering preventive interventions for maternal and child health in low- and middle-income countries: a systematic review. BMC Public Health. 2013;13:847. 10.1186/1471-2458-13-847 24034792 PMC3848754

[8] Perry HB Zulliger R Rogers MM Community Health Workers in Low-, Middle-, and High-Income Countries: An Overview of Their History, Recent Evolution, and Current Effectiveness. Annu Rev Public Health. 2014;35:399-421. 10.1146/annurev-publhealth-032013-182354 24387091

[9] World Health Organization. Scaling-up the Community-Based Health Workforce for Emergencies: Joint statement. Available: https://www.who.int/workforcealliance/knowledge/resources/chwstatement/en/Global Health Workforce. Accessed: 7 May 2021.

[10] Miller NP Richards AK Marx MA Checchi F Kozuki N Assessing community health worker service delivery in humanitarian settings. J Glob Health. 2020;10:010307. 10.7189/jogh.10.010307 32257135 PMC7100867

[11] Roome E Raven J Martineau T Human resource management in post-conflict health systems: review of research and knowledge gaps. Confl Health. 2014;8:18. 10.1186/1752-1505-8-18 25295071 PMC4187016

[12] Janneck L Cooper N Frehywot S Mowafi H Hein K Human Resources in Humanitarian Health Working Group Report. Prehosp Disaster Med. 2009;24:s184-93. 10.1017/S1049023X00021567 19806538

[13] Waters H, Garrett B, Burnham G. Rehabilitating health systems in post- conflict situations. In Making Peace Work: The Challenges of Social and Economic Reconstruction. New York: Palgrave Macmillan; 2009.

[14] Kozuki N Ericson K Marron B Lainez YB Miller NP The resilience of integrated community case management in acute emergency: a case study from Unity State, South Sudan. J Glob Health. 2018;8:020602. 10.7189/jogh.08.020602 30237877 PMC6119813

[15] Miller NP Ardestani FB Dini HS Shafique F Zunong N Community health workers in humanitarian settings: Scoping review. J Glob Health. 2020;10:020602. 10.7189/jogh.10.020602 33312508 PMC7719274

[16] Ruckstuhl L Lengeler C Moyen JM Garro H Allan R Malaria case management by community health workers in the Central African Republic from 2009–2014: overcoming challenges of access and instability due to conflict. Malar J. 2017;16:388. 10.1186/s12936-017-2005-7 28962622 PMC5622524

[17] Jaskiewicz W Tulenko K Increasing community health worker productivity and effectiveness: a review of the influence of the work environment. Hum Resour Health. 2012;10:38. 10.1186/1478-4491-10-38 23017131 PMC3472248

[18] Jain S The Role of Paraprofessionals in Providing Treatment for Posttraumatic Stress Disorder in Low-Resource Communities. JAMA. 2010;304:571-2. 10.1001/jama.2010.1096 20682940

[19] Miller NP Milsom P Johnson G Bedford J Kapeu AS Diallo AO Community health workers during the Ebola outbreak in Guinea, Liberia, and Sierra Leone. J Glob Health. 2018;8:020601. 30023054 10.7189/jogh-08-020601 PMC6030670

[20] Fredricks K Dinh H Kusi M Yogal C Karmacharya BM Burke TF Community Health Workers and Disasters: Lessons Learned from the 2015 Earthquake in Nepal. Prehosp Disaster Med. 2017;32:604-9. 10.1017/S1049023X1700680X 28786371

[21] Vallières F Hyland P McAuliffe E Mahmud I Tulloch O Walker P A new tool to measure approaches to supervision from the perspective of community health workers: a prospective, longitudinal, validation study in seven countries. BMC Health Serv Res. 2018;18:806. 10.1186/s12913-018-3595-7 30348147 PMC6196473

[22] Garrison K, Caiola N, Sullivan R. Supervising health care services: Improving the performance of people. Washington DC: JHPIEGO; 2004.

[23] Hill Z Dumbaugh M Benton L Källander K Strachan D ten Asbroek A Supervising community health workers in low-income countries – a review of impact and implementation issues. Glob Health Action. 2014;7:24085. 10.3402/gha.v7.24085 24815075 PMC4016747

[24] Jenkins R Othieno C Okeyo S Aruwa J Kingora J Jenkins B Health system challenges to integration of mental health delivery in primary care in Kenya- perspectives of primary care health workers. BMC Health Serv Res. 2013;13:368. 10.1186/1472-6963-13-368 24079756 PMC3852631

[25] Ledikwe JH Kejelepula M Maupo K Sebetso S Thekiso M Smith M Evaluation of a Well-Established Task-Shifting Initiative: The Lay Counselor Cadre in Botswana. PLoS One. 2013;8:e61601. 10.1371/journal.pone.0061601 23585912 PMC3621674

[26] Colvin CJ de Heer J Winterton L Mellenkamp M Glenton C Noyes J A systematic review of qualitative evidence on barriers and facilitators to the implementation of task-shifting in midwifery services. Midwifery. 2013;29:1211-21. 10.1016/j.midw.2013.05.001 23769757

[27] Kok MC Muula AS Motivation and job satisfaction of health surveillance assistants in Mwanza, Malawi: an explorative study. Malawi Med J. 2013;25:5-11. 23717748 PMC3653191

[28] Greenspan JA McMahon SA Chebet JJ Mpunga M Urassa DP Winch PJ Sources of community health worker motivation: a qualitative study in Morogoro Region, Tanzania. Hum Resour Health. 2013;11:52. 10.1186/1478-4491-11-52 24112292 PMC3852396

[29] Stekelenburg J Kyanamina SS Wolffers I Poor performance of community health workers in Kalabo District, Zambia. Health Policy. 2003;65:109-18. 10.1016/S0168-8510(02)00207-5 12849910

[30] Frimpong JA Helleringer S Awoonor-Williams JK Yeji F Phillips JF Does supervision improve health worker productivity? Evidence from the Upper East Region of Ghana. Trop Med Int Health. 2011;16:1225-33. 10.1111/j.1365-3156.2011.02824.x 21729221

[31] Ludwick T Turyakira E Kyomuhangi T Manalili K Robinson S Brenner JL Supportive supervision and constructive relationships with healthcare workers support CHW performance: Use of a qualitative framework to evaluate CHW programming in Uganda. Hum Resour Health. 2018;16:11. 10.1186/s12960-018-0272-1 29439743 PMC5812047

[32] Raven J Wurie H Idriss A Bah AJ Baba A Nallo G How should community health workers in fragile contexts be supported: qualitative evidence from Sierra Leone, Liberia and Democratic Republic of Congo. Hum Resour Health. 2020;18:58. 10.1186/s12960-020-00494-8 32770998 PMC7414260

[33] National Health Service. Clinical Outcomes. Available: https://gosh.nhs.uk/conditions-and-treatments/clinical-outcomes. Accessed: 4 October 2021.

[34] Peacock S, Chan C, Mangolini M, Johansen D. Techniques for Measuring Efficiency in Health Services, Productivity Commission Staff Working Paper; 2001.

[35] Olsen IT Sustainability of health care: a framework for analysis. Health Policy Plan. 1998;13:287-95. 10.1093/heapol/13.3.287 10187598

[36] World Health Organisation. Promoting Mental Health; Concepts emerging evidence and practice. Geneva: World Health Organisation; 2004.

[37] Covidence systematic review software. Veritas Health Innovation. Available: www.covidence.org. Accessed: 15 March 2021.

[38] Sutherland SE An Introduction to Systematic Reviews. J Evid Based Dent Pract. 2004;4:47-51. 10.1016/j.jebdp.2004.02.021

[39] Critical Appraisal Skills Programme. CASP Qualitative Studies Checklist. Available: https://casp-uk.net/casp-tools-checklists/. Accessed: 5 August 2021.

[40] Critical Appraisal Skills Programme. CASP Randomised Controlled Trials Checklist. Available: https://casp-uk.net/casp-tools-checklists/. Accessed: 5 August 2021.

[41] Rahman A Akhtar P Hamdani SU Atif N Nazir H Uddin I Using technology to scale-up training and supervision of community health workers in the psychosocial management of perinatal depression: a non-inferiority, randomized controlled trial. Glob Ment Health (Camb). 2019;6:e8. 10.1017/gmh.2019.7 31157115 PMC6533850

[42] Miller NP Zunong N Al-Sorouri TAA Alqadasi YM Ashraf S Siameja C Implementing integrated community case management during conflict in Yemen. J Glob Health. 2020;10:020601. 10.7189/jogh.10.020601 33110596 PMC7568935

[43] Horn R O’May F Esliker R Gwaikolo W Woensdregt L Ruttenberg L The myth of the 1-day training: the effectiveness of psychosocial support capacity-building during the Ebola outbreak in West Africa. Glob Ment Health (Camb). 2019;6:e5. 10.1017/gmh.2019.2 31143466 PMC6521134

[44] McLean KE Kaiser BN Hagaman AK Wagenaar BH Therosme TP Kohrt BA Task sharing in rural Haiti. Intervention (Amstelveen). 2015;13:135-55. 10.1097/WTF.0000000000000074 26190953 PMC4501397

[45] Wong JHC Leung CTL Trauma-informed practice and supervision for volunteer counsellors of online psychological support groups during the impact of COVID-19. Asia Pac J Soc Work Dev. 2020;31:67-72. 10.1080/02185385.2020.1846604

[46] Moola S, Munn Z, Tufanaru C, Aromataris E, Sears K, Sfetcu R, et al. Chapter 7: Systematic reviews of etiology and risk. Available: https://reviewersmanual.joannabriggs.org/. Accessed: 5 May 2021.

[47] Aldamman K Tamrakar T Dinesen C Wiedemann N Murphy J Hansen M Caring for the mental health of humanitarian volunteers in traumatic contexts: the importance of organisational support. Eur J Psychotraumatol. 2019;10:1694811. 10.1080/20008198.2019.1694811 31839900 PMC6896515

[48] Hong QN, Pluye P, Fàbregues S, Bartlett G, Boardman F, Cargo M, et al. Mixed Methods Appraisal Tool (MMAT). Registration of Copyright (#1148552), Canadian Intellectual Property Office, Industry Canada; 2018.

[49] Murray LK Dorsey S Haroz E Lee C Alsiary MM Haydary A A Common Elements Treatment Approach for Adult Mental Health Problems in Low- and Middle-Income Countries. Cogn Behav Pract. 2014;21:111-23. 10.1016/j.cbpra.2013.06.005 25620867 PMC4304666

[50] Magidson JF Lejuez CW Kamal T Blevins EJ Murray LK Bass JK Adaptation of community health worker-delivered behavioral activation for torture survivors in Kurdistan, Iraq. Glob Ment Health (Camb). 2015;2:e24. 10.1017/gmh.2015.22 27478619 PMC4962865

[51] Shah R Miller NP Mothabbir G Approaches to support continued iCCM implementation during a flooding emergency in rural Bangladesh. J Glob Health. 2019;9:021001. 10.7189/jogh.09.021001 31893038 PMC6925969

[52] Page MJ McKenzie JE Bossuyt PM Boutron I Hoffmann TC Mulrow CD The PRISMA 2020 statement: an updated guideline for reporting systematic reviews. BMJ. 2021;372. 33782057 10.1136/bmj.n71 PMC8005924

[53] Kohrt BA Ramaiya MK Rai S Bhardwaj A Jordans MJD Development of a scoring system for non-specialist ratings of clinical competence in global mental health: a qualitative process evaluation of the Enhancing Assessment of Common Therapeutic Factors (ENACT) scale. Glob Ment Health (Camb). 2015;2:e23. 10.1017/gmh.2015.21 28593049 PMC5269630

[54] Murray LK Dorsey S Bolton P Jordans MJ Rahman A Bass J Building capacity in mental health interventions in low resource countries: an apprenticeship model for training local providers. Int J Ment Health Syst. 2011;5:30. 10.1186/1752-4458-5-30 22099582 PMC3284435

[55] van der Veer G de Jong K Lansen J Clinical supervision for counsellors in areas of armed conflict. Intervention (Amstelveen). 2004;2:118-29.

[56] World Health Organization. Training for mid-level managers (MLM) Module 4: Supportive supervision. Available: https://www.who.int/immunization/documents/MLM_module4.pdf?ua=1. Accessed: 5 May 2021.

[57] Travers A, Abujaber N, McBride K, Blum P, Wiedemann N, Vallières F. Identifying best practice for the supervision of mental health and psychosocial support in humanitarian emergencies: A Delphi study. Forthcoming. 10.1186/s13033-022-00515-0 PMC8822743 35130947

