# Supplementary material for: Examining the evidence for best practice guidelines in supportive supervision of lay health care providers in humanitarian emergencies: A systematic scoping review
Source: J Glob Health. 2022 Feb 27;12:04017. doi: 10.7189/jogh.12.04017 (PMC8876157; doi:10.7189/jogh.12.04017)
Supplement: Online Supplementary Document [file jogh-12-04017-s001.pdf]

Web of Science 11-01-21 1315

Search in Topic

(volunteer* OR "nonspecialist*" OR "non specialist*" OR "unlicensed provider*" OR "non professional*" OR nonprofessional* OR "para professional*" OR paraprofessional* OR "birth attendant*" OR assistant* OR trainee* OR healer* OR aide* OR "CHW" OR "psychosocial support" OR counsellor* OR counselor* OR "MHPSS" OR "Thinking Healthy" OR "Psychological First Aid" OR "mhGAP" OR "self help" OR "Problem Management Plus" OR "PM+" OR ((traditional OR complementary OR alternative) **NEAR/2** medicine) OR ((healthcare OR health) **NEAR/2** (primary OR worker* OR educator*)) OR ((lay OR untrained OR "lesser trained" OR "non clinical" OR "non medical") **NEAR/2** (advisor* OR staff* OR worker* OR provider*)))

AND

(supervis* OR mentor* OR apprentic* OR coach* OR oversee* OR "task shifting" OR "task sharing" OR (capacity **NEAR/2** build*))

AND

(humanitarian OR disaster* OR war OR wars OR warzone* OR militant* OR "post conflict" OR emergenc* OR crisis OR crises OR tsunami* OR earthquake* OR typhoon* OR storm* OR hurricane* OR refugee* OR survivor* OR "displaced persons" OR Ebola OR tortur* OR ((armed OR political) **NEAR/2** (conflict* OR violence OR attack*)))

Limit to English

EMBASE (NOT MEDLINE) 11/01/21 943

('volunteer'/exp OR 'voluntary worker'/exp OR (volunteer* OR "nonspecialist*" OR "non specialist*" OR "unlicensed provider*" OR "non professional*" OR nonprofessional* OR "para professional*" OR paraprofessional* OR "birth attendant*" OR assistant* OR trainee* OR healer* OR aide* OR "CHW" OR "psychosocial support" OR counsellor* OR counselor* OR "MHPSS" OR "Thinking Healthy" OR "Psychological First Aid" OR "mhGAP" OR "self help" OR "Problem Management Plus" OR "PM+" OR ((traditional OR complementary OR alternative) **NEAR/2** medicine) OR ((healthcare OR health) **NEAR/2** (primary OR worker* OR educator*)) OR ((lay OR untrained OR "lesser trained" OR "non clinical" OR "non medical") **NEAR/2** (advisor* OR staff* OR worker* OR provider*))):ab,ti,kw)

AND

('mentor'/exp OR 'mentoring'/exp OR (supervis* OR mentor* OR apprentic* OR coach* OR oversee* OR "task shifting" OR "task sharing" OR (capacity **NEAR/2** build*)):ab,ti,kw)

AND

('disaster'/exp OR 'disaster victim'/exp OR 'severe weather'/exp OR 'war'/exp OR 'migrant'/exp OR 'survivor'/exp OR (humanitarian OR disaster* OR war OR wars OR warzone* OR militant* OR "post conflict" OR emergenc* OR crisis OR crises OR tsunami* OR earthquake* OR typhoon* OR storm* OR hurricane* OR refugee* OR survivor* OR "displaced persons" OR Ebola OR tortur* OR ((armed OR political) **NEAR/2** (conflict* OR violence OR attack*))):ab,ti,kw) NOT [MEDLINE]/lim AND [english]/lim

Limit to English

MEDLINE 11-01-21 1034

(MH "Volunteers+" OR TI(volunteer* OR "nonspecialist*" OR "non specialist*" OR "unlicensed provider*" OR "non professional*" OR nonprofessional* OR "para professional*" OR paraprofessional* OR "birth attendant*" OR assistant* OR trainee* OR healer* OR aide* OR "CHW" OR "psychosocial support" OR counsellor* OR counselor* OR "MHPSS" OR "Thinking Healthy" OR "Psychological First Aid" OR "mhGAP" OR "self help" OR "Problem Management Plus" OR "PM+" OR ((traditional OR complementary OR alternative) **N2** medicine) OR ((healthcare OR health) **N2** (primary OR worker* OR educator*)) OR ((lay OR untrained OR "lesser trained" OR "non clinical" OR "non medical") **N2** (advisor* OR staff* OR worker* OR provider*))) OR AB(volunteer* OR "nonspecialist*" OR "non specialist*" OR "unlicensed provider*" OR "non professional*" OR nonprofessional* OR "para professional*" OR paraprofessional* OR "birth attendant*" OR assistant* OR trainee* OR healer* OR aide* OR "CHW" OR "psychosocial support" OR counsellor* OR counselor* OR "MHPSS" OR "Thinking Healthy" OR "Psychological First Aid" OR "mhGAP" OR "self help" OR "Problem Management Plus" OR "PM+" OR ((traditional OR complementary OR alternative) **N2** medicine) OR ((healthcare OR health) **N2** (primary OR worker* OR educator*)) OR ((lay OR untrained OR "lesser trained" OR "non clinical" OR "non medical") **N2** (advisor* OR staff* OR worker* OR provider*))))

AND

(MH "Mentors" OR MH "Mentoring" OR TI(supervis* OR mentor* OR apprentic* OR coach* OR oversee* OR "task shifting" OR "task sharing" OR (capacity **N2** build*)) OR AB(supervis* OR mentor* OR apprentic* OR coach* OR oversee* OR "task shifting" OR "task sharing" OR (capacity **N2** build*)))

AND

(MH "Disasters+" OR MH "Warfare and Armed Conflicts+" OR MH "Transients and Migrants" OR MH "Survivors+" OR TI(humanitarian OR disaster* OR war OR wars OR warzone* OR militant* OR "post conflict" OR emergenc* OR crisis OR crises OR tsunami* OR earthquake* OR typhoon* OR storm* OR hurricane* OR refugee* OR survivor* OR "displaced persons" OR Ebola OR tortur* OR ((armed OR political) **N2** (conflict* OR violence OR attack*))) OR AB(humanitarian OR disaster* OR war OR wars OR warzone* OR militant* OR "post conflict" OR emergenc* OR crisis OR crises OR tsunami* OR earthquake* OR typhoon* OR storm* OR hurricane* OR refugee* OR survivor* OR "displaced persons" OR Ebola OR tortur* OR ((armed OR political) **N2** (conflict* OR violence OR attack*))))

Limit to English

CINAHL (NOT MEDLINE) 11-01-21 300

(MH "Volunteer Experiences" OR MH "Volunteer Workers" OR MH "Community Service" OR TI(volunteer* OR "nonspecialist*" OR "non specialist*" OR "unlicensed provider*" OR "non professional*" OR nonprofessional* OR "para professional*" OR paraprofessional* OR "birth attendant*" OR assistant* OR trainee* OR healer* OR aide* OR "CHW" OR "psychosocial support" OR counsellor* OR counselor* OR "MHPSS" OR "Thinking Healthy" OR "Psychological First Aid" OR "mhGAP" OR "self help" OR "Problem Management Plus" OR "PM+" OR ((traditional OR complementary OR alternative) **N2** medicine) OR ((healthcare OR health) **N2** (primary OR worker* OR educator*)) OR ((lay OR untrained OR "lesser

trained" OR "non clinical" OR "non medical") **N2** (advisor* OR staff* OR worker* OR provider*))) OR AB(volunteer* OR "nonspecialist*" OR "non specialist*" OR "unlicensed provider*" OR "non professional*" OR nonprofessional* OR "para professional*" OR paraprofessional* OR "birth attendant*" OR assistant* OR trainee* OR healer* OR aide* OR "CHW" OR "psychosocial support" OR counsellor* OR counselor* OR "MHPSS" OR "Thinking Healthy" OR "Psychological First Aid" OR "mhGAP" OR "self help" OR "Problem Management Plus" OR "PM+" OR ((traditional OR complementary OR alternative) **N2** medicine) OR ((healthcare OR health) **N2** (primary OR worker* OR educator*)) OR ((lay OR untrained OR "lesser trained" OR "non clinical" OR "non medical") **N2** (advisor* OR staff* OR worker* OR provider*))))

AND

(MH "Mentorship" OR TI(supervis* OR mentor* OR apprentic* OR coach* OR oversee* OR "task shifting" OR "task sharing" OR (capacity **N2** build*)) OR AB(supervis* OR mentor* OR apprentic* OR coach* OR oversee* OR "task shifting" OR "task sharing" OR (capacity **N2** build*)))

AND

(MH "Disasters+" OR MH "War+" OR MH "Transients and Migrants" OR MH "Survivors+" OR TI(humanitarian OR disaster* OR war OR wars OR warzone* OR militant* OR "post conflict" OR emergenc* OR crisis OR crises OR tsunami* OR earthquake* OR typhoon* OR storm* OR hurricane* OR refugee* OR survivor* OR "displaced persons" OR Ebola OR tortur* OR ((armed OR political) **N2** (conflict* OR violence OR attack*))) OR AB(humanitarian OR disaster* OR war OR wars OR warzone* OR militant* OR "post conflict" OR emergenc* OR crisis OR crises OR tsunami* OR earthquake* OR typhoon* OR storm* OR hurricane* OR refugee* OR survivor* OR "displaced persons" OR Ebola OR tortur* OR ((armed OR political) **N2** (conflict* OR violence OR attack*))))

Limit to English

PsycInfo 11-01-21 592

(DE "Volunteers" OR TI(volunteer* OR "nonspecialist*" OR "non specialist*" OR "unlicensed provider*" OR "non professional*" OR nonprofessional* OR "para professional*" OR paraprofessional* OR "birth attendant*" OR assistant* OR trainee* OR healer* OR aide* OR "CHW" OR "psychosocial support" OR counsellor* OR counselor* OR "MHPSS" OR "Thinking Healthy" OR "Psychological First Aid" OR "mhGAP" OR "self help" OR "Problem Management Plus" OR "PM+" OR ((traditional OR complementary OR alternative) **N2** medicine) OR ((healthcare OR health) **N2** (primary OR worker* OR educator*)) OR ((lay OR untrained OR "lesser trained" OR "non clinical" OR "non medical") **N2** (advisor* OR staff* OR worker* OR provider*))) OR AB(volunteer* OR "nonspecialist*" OR "non specialist*" OR "unlicensed provider*" OR "non professional*" OR nonprofessional* OR "para professional*" OR paraprofessional* OR "birth attendant*" OR assistant* OR trainee* OR healer* OR aide* OR "CHW" OR "psychosocial support" OR counsellor* OR counselor* OR "MHPSS" OR "Thinking Healthy" OR "Psychological First Aid" OR "mhGAP" OR "self help" OR "Problem Management Plus" OR "PM+" OR ((traditional OR complementary OR alternative) **N2** medicine) OR ((healthcare OR health) **N2** (primary OR worker* OR educator*)) OR ((lay OR untrained OR "lesser trained" OR "non clinical" OR "non medical") **N2** (advisor* OR staff* OR worker* OR provider*))))

AND

(DE "Mentor" OR TI(supervis* OR mentor* OR apprentic* OR coach* OR oversee* OR "task shifting" OR "task sharing" OR (capacity **N2** build*)) OR AB(supervis* OR mentor* OR apprentic* OR coach* OR oversee* OR "task shifting" OR "task sharing" OR (capacity **N2** build*)))

AND

(DE "Disasters" OR DE "Natural Disasters" OR DE "Emergency Preparedness" OR DE "Emergency Management" OR DE "Emergency Preparedness" OR DE "Survivors" OR DE "Holocaust Survivors" OR DE "Symbolic Interactionism" OR DE "War" OR DE "Nuclear War" OR DE "Refugees" OR TI(humanitarian OR disaster* OR war OR wars OR warzone* OR militant* OR "post conflict" OR emergenc* OR crisis OR crises OR tsunami* OR earthquake* OR typhoon* OR storm* OR hurricane* OR refugee* OR survivor* OR "displaced persons" OR Ebola OR tortur* OR ((armed OR political) **N2** (conflict* OR violence OR attack*))) OR AB(humanitarian OR disaster* OR war OR wars OR warzone* OR militant* OR "post conflict" OR emergenc* OR crisis OR crises OR tsunami* OR earthquake* OR typhoon* OR storm* OR hurricane* OR refugee* OR survivor* OR "displaced persons" OR Ebola OR tortur* OR ((armed OR political) **N2** (conflict* OR violence OR attack*))))

Limit to English

PDAT 11-01-21 443

(volunteer* OR "nonspecialist*" OR "non specialist*" OR "unlicensed provider*" OR "non professional*" OR nonprofessional* OR "para professional*" OR paraprofessional* OR "birth attendant*" OR assistant* OR trainee* OR healer* OR aide* OR "CHW" OR "psychosocial support" OR counsellor* OR counselor* OR "MHPSS" OR "Thinking Healthy" OR "Psychological First Aid" OR "mhGAP" OR "self help" OR "Problem Management Plus" OR "PM+" OR ((traditional OR complementary OR alternative) **NEAR/2** medicine) OR ((healthcare OR health) **NEAR/2** (primary OR worker* OR educator*)) OR ((lay OR untrained OR "lesser trained" OR "non clinical" OR "non medical") **NEAR/2** (advisor* OR staff* OR worker* OR provider*)))

AND

(supervis* OR mentor* OR apprentic* OR coach* OR oversee* OR "task shifting" OR "task sharing" OR (capacity **NEAR/2** build*))

AND

(humanitarian OR disaster* OR war OR wars OR warzone* OR militant* OR "post conflict" OR emergenc* OR crisis OR crises OR tsunami* OR earthquake* OR typhoon* OR storm* OR hurricane* OR refugee* OR survivor* OR "displaced persons" OR Ebola OR tortur* OR ((armed OR political) **NEAR/2** (conflict* OR violence OR attack*)))

Limit to anywhere but full text
